# Choice of Treatment Used in a Patient With Antipsychotic-Induced Rhabdomyolysis

**DOI:** 10.1192/bjo.2025.10179

**Published:** 2025-06-20

**Authors:** Mizanoor Rahman, Risha Ruparelia

**Affiliations:** North London NHS Foundation Trust, London, United Kingdom

## Abstract

**Aims:** Raised creatine kinase (CK) secondary to antipsychotics is often discussed in the context of neuroleptic malignant syndrome (NMS). However, it is documented that antipsychotic-induced CK can also result from rhabdomyolysis, with limited data available on the risk profile of specific antipsychotics.

**Methods:** We report the case of a 42-year-old woman with paranoid schizophrenia, maintained on olanzapine for years and recently started on a combination of olanzapine and lurasidone. She was admitted to an intensive care unit following seizures and severe hyponatraemia (sodium level 113). Both antipsychotics were stopped initially due to concerns about their role in hyponatraemia; later identified as secondary to psychogenic polydipsia. Upon olanzapine reintroduction, CK levels rose from 9,000 to 32,000 overnight, prompting immediate discontinuation. As there were no NMS symptoms, olanzapine was reintroduced but subsequently stopped again after CK levels peaked at 77,000 and liver function tests deteriorated.

The patient was reviewed by Rheumatology, who suggested olanzapine-induced eosinophilic myositis and rhabdomyolysis. This resulted in the patient developing compartment syndrome; hence a slower CK decline, and bilateral foot drop which was reflected on the nerve conduction studies.

Steroids were initiated for compartment syndrome, and antipsychotics were withheld until CK normalised. The patient was commenced on risperidone, but within a few days the CK increased to 1,000, necessitating its discontinuation. Aripiprazole was then trialled, but the CK rose to 737 after three doses and it was therefore ceased. Benzodiazepines were temporarily used to manage emerging psychotic symptoms until CK levels stabilised. The patient was then transferred to an inpatient psychiatric ward. Given CK elevation with three atypical antipsychotics, a typical antipsychotic, namely haloperidol, was cautiously introduced. It was successfully titrated to a therapeutic dose without CK elevation.

**Results:** The case recognises the potential occurrence of rhabdomyolysis secondary to antipsychotics and the medical complications as a result. It underscores the importance of close monitoring of CK when prescribing antipsychotics. More importantly, a pattern of atypical antipsychotics being the key factor for rhabdomyolysis was identified. Thus, trialling a typical antipsychotic could be beneficial in treating psychotic disorders in such cases.

**Conclusion:** This case has identified that typical antipsychotics may have a lower risk of causing rhabdomyolysis compared with atypical antipsychotics, but the mechanism behind this is unclear. This should be kept in mind, particularly in patients with a history of elevated CK who require treatment for their mental health.

